# Personalised iron supply for prophylaxis and treatment of pregnant women as a way to ensure normal iron levels in their breast milk

**Published:** 2012-03-05

**Authors:** GH Marin, N Mestorino, J Errecalde, B Huber, A Uriarte, J Orchuela

**Affiliations:** Faculty of Medical Sciences, National University, La Plata. Argentina; Public Health Service, La Plata Buenos Aires, Argentina

**Keywords:** breastfeeding, iron deficiency, health, policy

## Abstract

**Rationale: **Because the characteristics of all body fluids depends on patient’s health status, is it possible that disadvantaged and socially vulnerable mothers may have lower amounts of iron in their breast milk, and that their babies receive lower content of the mineral for their normal growth and development. Assuring a preventive treatment of the mother might solve this problem.

**Objective: **to demonstrate breast milk iron content from disadvantaged mothers and impact of personalized iron supplementation program.

**Materials and Methods: **cross-sectional study. Breast milk samples were obtained for ferritin analysis. Health’s services usually provides free folic acid and iron treatment however, treatment compliance is low. Patients were random in two groups: “A: Controls” that had free iron tablets available from Health Centre; and “B: Intervention” group where patients accepted to be periodically contacted at home by health’s team for personalized iron dispensation.

**Results: **360 patients were included. Profilaxis and treatment compliance were 100% and 97,6% for B group while for “Control” one was 63% and 34%(p0.0001). Higher breast milk iron levels were detected in Intervention’s mothers compared with control’s patients (p0.007).

**Conclusion: **Personalized iron prophylaxis and treatment increased breast milk iron levels. Public health policy must ensure iron dispensation for each underserved mother in order to reduce children problems associate to iron deficiency during the first year of their life.

## Introduction

Iron deficiency is one of the major health problems among children less than 1 year old [**1**]. Maternal anaemia leads to anaemia in newborn infants [**2**]. Unfortunately, iron deficiency and anaemia are prevalent in many countries of South America, associated to the socio-economical situation [**3**]. 

Iron deficiency can have deleterious effects on behaviour [**4-7**], immune system [**8-13**], and mental [**5**] and physical [**14**] development. Prevention of anaemia in newborns should therefore be a priority for public health policies.

Although substantial prevalence rates among disadvantaged children have been reported [**4**], official programs attempted to correct this iron deficiency anaemia by delivering iron-fortified milk and other dietary supplements available free of charge to the underprivileged population in many countries [**5**].

However, this point is controversial since even if official policies enhance the breastfeeding during the first 6 months of life of newborns [**15**], the quality of that breast milk will depend on the mother’s health and nutrition. 

We started the present study, in order to demonstrate the iron content of breast milk from mothers belonging to disadvantaged families and the impact of a personalized program for iron suplementation on those mothers.

## Methods

### Type of Study and Population sample

This is a cross-sectional study consisting of two phases (descriptive and analytical) with a quantitative approach. This study was conducted in the capital of Buenos Aires province (La Plata) which, according to the National Institute of Statistics and Census (INDEC) (2001 census) is a faithful exponent of most Argentinian urban cities [**16**]. 

Breast milk samples were obtained from pregnant women belonging to disadvantaged families defined by the household income, geographical proximity of the house surveyed to a local health center (LHC) and by the Unsatisfed Basic Needs (UBN) index [**17**], which is an index composed of socio-economic indicators incorporating like house overcrowding (three or more family members per room); lack of sewerage services; unstable house construction; illiteracy in at least one member of that family; or children in the house who is not attending school. 

The sample size was calculated according to routine control theory, with a confidence of 95% (alpha error = 5%) and an accuracy of 5% in a theoretical minimum of 124 dwellings, using the following formula:

. N • Z2 • p (1-p) 

n = ---------------------------

. d2 • (N-1) + Z2 • p (1-p) 

where n is the sample size, N the total population, Z the value of z for confidence level (1 - alpha), p the expected proportion of iron deficiency in the population and d the absolute accuracy. 

The unit of analysis was the “pregnant women”. The criteria for including participants in the study were: age (mothers who at the time of the survey were 16 years or older); pregnant women coming from the geographical area selected for the study or that lived in the region for over a year; and that voluntary accepted to participate in the study (consent agreement).

Before milk sample collection, a second inclusion criteria was applied to all mothers previously included in the study. These criteria were: 1) gestational age ≥ 37 weeks, 2) birth weight > 2500 grams 3) infant exclusively breastfed at 2 months.

### Groups of Study

For all pregnant women, folic acid (5 mg folate pill/day) and iron (200mg pill of ferrous sulphate once or twice day) treatment was available. However, sample was random divided into two groups: Group A received free medicines from the Health Center near their homes; Group B accepted to be contacted monthly by a member of the Health team in order to personalized free iron dispense for prophylaxis or treatment during all pregnancy period until 45 days after the delivery. This medicine was always dispensed by that same health professional either through the health center, or in case that patient did not attend his periodical consultation, drugs were dispensed at the patient’s home. 

### Milk sampling and laboratory assay

For sample collection, mothers were instructed to ﬁrst clean their breasts with a sterile disposable towel soaked with water, then allowing the area to air dry. Breast-milk samples were collected at 2nd month postpartum. Samples were collected during the morning after 2 hours after the previous breastfeeding. Milk expression was done from only one breast, between from 9:00 am to 11:00 am under supervision of her Obstetric. All mothers received explicit instructions regarding the handling of a glass jar. After discarding an initial 4 mL of milk, an aliquot of 10 ml was collected by manual plastic disposable pump into clean sterile tested plastic jars containers provided by the National University of La Plata (UNLP) and transferred to iron-free polyethylene tubes. Milk samples were preserved at 4°C until they were analyzed for iron content by absorption spectrophotometry. None of the mothers had mastitis or febrile illness during the period of study. 

Samples were ﬁnally transferred to screw-cap, plastic tubes and frozen at -20 °C until analysis. For laboratory iron analysis, samples were thawed, mixed thoroughly, and processed with a trienzyme digestion procedure before microbiological assay at room temperature and then at subboiling temperature for 6–9 h. Iron concentration was determined by absorption spectrometry. The analyzed reference values were within 95–105% of the certified value.

### Statistical analysis

Student’s t test was used for variables analysis with was performed by using statistical software SPSS 10. Statistical significance was set at P < 0.05. All values in the text are expressed as means ± SDs

## Results

Three hundred and sixty patients were included in the study. 174 of them were included in group A (control) and 186 patients were followed up in a personalized iron treatment (group B – intervention). 

Both study groups (A control, B intervention) were comparable in maternal age, maternal median serum hemoglobin level, gestational age and new born birth weight (**Table 1**). 

**Table 1:** Patient’s features

Parameter	Group A (control n=174)	Group B (personalized medicine dispense n=186)	P value
maternal age (minutes)	26,93± 7.41	27,43± 6.07	NS
median serum hemoglobin level (gr/dl)	11,41 ± 1,02	11,79 ± 1,42	NS
gestational age (weeks)	39±4.5	39±7.2	NS
newborn weight	3301±574	3309±611	NS
time to cut the umbilical cord (minutes)	3.9±2.1	3.7±1.8	NS
NS: non significant			

Free iron tablets (each tablet: 200 mg of iron sulphate -60mg of elemental iron-) and folic acid tablets (each tablet: 1 mg of folate) were available all time in health centres (HC). 

Any health team member could give free iron/folate prophylaxis or treatment to the patients, either if they demanded so (group A –“control group”) or if patients received medicines in a personalized dispensed way by their responsible person in health centre or at home (group B). In this last case, Public health service labelled the medicines containers with each patient’s name and last name. 

Iron and Folate prophylaxis was given to a 100% of mothers belonging to Intervention group (group B) while in anaemia’s patients, treatment compliance was 97,6% for the same group. For Control Group “A”, prophylaxis and treatment compliance were 63% and 34% respectively (statistical differences between A and B groups were p 0.003 and 0.0001 for prophylaxis and treatment respectively). 

Comparing control’s mothers (group A), with mothers that received personalized treatment during their pregnancy (group B) it was observed that the last group had higher iron levels in their breast milk (**Table 2**), (**Table 3**).

**Table 2:** Iron levels in anaemic patients during their last trimester of pregnancy

Parameter	Group A* (n=81 )	Group B** (n=59 )	P value
Iron milk levels (mg/L)	0.29 ± 0.18	0.41 ±0.15	0.007
*Controls patients ; ** Intervention group			

**Table 3:** Breast milk iron levels

Parameter	Group A (control n=174)	Group B (personalized medicine dispense n=186)	P value
Iron milk levels (mg/L)	0.32 ± 0.14	0.48 ±0.17	< 0.001

Even in mothers who had anaemia during their last trimester of pregnancy, personalized regular iron dispense, assured higher breast milk iron levels than the ones of the control group. 

## Discussion

Iron deficiency is frequent in children under 1 year old in many developing countries like Argentina. Assuring iron prophilaxis and treatment to mothers during the pregnancy period may reduce this health problem. 

Especially in Argentina, the access to iron treatment is guaranteed by the Ministry of Health, by REMEDIAR program, which provides the Health Centres enough iron for all population. However, although medicine is available, treatment is still not assured since patients must have access to health centres to obtain the treatment.

We have previously demonstrated this situation [**18**], providing a medicine is not enough; an alternative strategy to obtain better treatment compliance is then needed. 

Personalized attention might improve health care performance as our group has shown previously [**19**]. 

It is well known that severe anaemia, present in the early gestation period, along with concomitant maternal malnutrition, may be associated with anaemia in newborns. 

Nowadays, because of the convenience of mothers, commercial’s milk formulations have been used (and abused) to feed their newborns. However, official recommendations are in favor of maintaining exclusive breastfeeding until six months of life, as it does clearly provide immunological benefits for the children.

It is therefore extremely important to ensure the best possible quality and highest iron content as possible of breast milk.

The present study demonstrated that iron content in breast milk is impaired when mothers have iron-deﬁciency during the pregnancy period. Even if mothers had anaemia during pregnancy, our data shows that a program that assures a personalized iron treatment, improves breast milk iron content.

An official program that guarantees iron prophylaxis and treatment to pregnant women will increase breast milk iron levels reducing children problems associate to iron deficiency during the first year of their life. 

The authors have no conflict of interest to declare about this paper. All the funds for this study were provided by the National University of La Plata, Argentina.

## References

[1] Chaparro  CM (2008). Setting the stage for child health and development: prevention of iron deficiency in early infancy. J Nutr.

[2] Marin  GH, Rivadulla  P, Negro  L (2008). Population study of the prevalence of anaemia in the adult population of Buenos Aires, Argentina. Aten Primaria.

[3] Marin  GH, Fazio  P, Rubbo  S (2002). Prevalence of anaemia in pregnancy and analysis of the underlying factors. Aten Primaria.

[4] Oppenheimer  SJ (2001). Iron and its relation to immunity and infectious disease. J Nutr.

[5] Lozoff  B (2011). Early iron deficiency has brain and behavior effects consistent with dopaminergic dysfunction. J Nutr.

[6] Chang  S, Wang  L, Wang  Y (2011). Iron-deficiency anaemia in infancy and social emotional development in preschool-aged Chinese children. Pediatrics.

[7] Hernández-Martínez  C, Canals  J, Aranda  N (2011). Effects of iron deficiency on neonatal behavior at different stages of pregnancy. Early Hum Dev.

[8] Muñoz  C, Rios  E, Olivos  J (2007). Iron, copper and immunocompetence. Br J Nutr.

[9] Wintergerst  ES, Maggini  S, Hornig  DH (2007). Contribution of selected vitamins and trace elements to immune function. Ann Nutr Metab.

[10] Cunningham-Rundles S, McNeeley  DF, Moon  A (2005). Mechanisms of nutrient modulation of the immune response. J Allergy Clin Immunol.

[11] Ahluwalia  N, Sun  J, Krause  D (2004). Immune function is impaired in iron-deficient, homebound, older women. Am J Clin Nutr.

[12] Rivera  MT, De Souza  AP, Araujo-Jorge TC (2003). Trace elements, innate immune response and parasites. Clin Chem Lab Med.

[13] Lönnerdal B (2009). Nutritional roles of lactoferrin. Curr Opin Clin Nutr Metab Care.

[14] Madan  N, Rusia  U, Sikka  M (2011). Developmental and neurophysiologic deficits in iron deficiency in children. Indian J Pediatr.

[15] UNICEF Breast feed.

[16] Indec  NBI, Etchegoyen  G, Paganini  J (2007). The relationship between socioeconomic factors and health programs in Argentine provinces. Rev Panam Salud Pública.

[17] INDEC Population Data.

[18] Marin  GH, Cañas  M, Homar  C (2008). Usage of Drug Prescriptions through the REMEDIAR Program in the Province of Buenos Aires, Argentina. . Lat. Am. J. Pharm.

[19] Marin  GH, Silberman  M, Etchegoyen  G (2008). A personalised health care programme (PANDELAS) operating in Buenos Aires, Argentina, during 2006. Rev. Salud Pública.

